# Nasal Provocation Test with Cat and Dog Extracts: Results according to Molecular Components

**DOI:** 10.1155/2020/6365314

**Published:** 2020-01-24

**Authors:** Andres Sánchez, Ricardo Cardona, Marlon Munera, Victor Calvo, Manuela Tejada-Giraldo, Jorge Sánchez

**Affiliations:** ^1^Group of Clinical and Experimental Allergy, Clinic “IPS Universitaria”, University of Antioquia, Medellín, Colombia; ^2^Medicine Department, University Corporation, Rafael Nuñez, Cartagena, Colombia; ^3^Foundation for the Development of Medical and Biological Sciences, Cartagena, Colombia

## Abstract

**Background:**

IgE sensitization (atopy) to pets is commonly evaluated using pet dander extracts. However, the diagnosis by components seems to be more adequate to evaluate the clinical relevance (allergy) of sIgE sensitization.

**Objective:**

To study the association between IgE sensitization to pet allergen components and clinical symptoms. *Methodology*. Dander extracts and sIgE levels to pet components (Can f 1, Can f 2, Can f 3, Can f 5, Fel d 1, Fel 2, and Fel 4) were measured in a rhinitis group (*n* = 101) and a control group (*n* = 101) and a control group (

**Results:**

Dog (34.6% vs. 23.5%) and cat dander (26.7% vs. 8.8%, *p* = 0.05) IgE sensitization was frequent among rhinitis and no-rhinitis subjects, and it was similar to dog (29.7% vs. 20.5%) and cat (18.8% vs. 8.8%) components. Polysensitization for dog (3.1, 95% CI: 1.5 to 6.1, *p* = 0.05) IgE sensitization was frequent among rhinitis and no-rhinitis subjects, and it was similar to dog (29.7% vs. 20.5%) and cat (18.8% vs. 8.8%) components. Polysensitization for dog (3.1, 95% CI: 1.5 to 6.1, *p* = 0.05) IgE sensitization was frequent among rhinitis and no-rhinitis subjects, and it was similar to dog (29.7% vs. 20.5%) and cat (18.8% vs. 8.8%) components. Polysensitization for dog (3.1, 95% CI: 1.5 to 6.1,

**Conclusions:**

Sensitization to pet dander extract identifies atopic patients, but its utility to predict clinical relevance is poor. Allergenic components could help to define the clinical relevance of sensitization to furry animals and could reduce the need for provocation test.

## 1. Introduction

Atopy to pets is considered an important risk factor for respiratory allergic diseases [1, 2]. According to GA^2^LEN [3], sIgE sensitization to pets, especially cats (24.8% to 27.9%) and dogs (25.6% to 28.8%), is very common among rhinitis patients in Europe, North America, and South America [4, 5]. The high sIgE sensitization to pets could be explained by the increasing exposure to cats and dogs in homes especially in urban cities [6, 7], but other investigations suggest that close contact with some pets prevents the development of allergy diseases [8].

Although there is extensive information about the prevalence of atopy to pet dander, there are still questions that merits particular analysis. Similar to what happens with allergy to food or pollen grains [9, 10], several studies based in patient's self-report suggest a relationship between furry animal allergen components and the risk of asthma, rhinitis, and the severity of these allergic diseases [11–13], but few studies verify this association with objective measures. The aim of this study was to evaluate pet dander extract and molecular components from cats and dogs, as specific markers of clinical response according to the results of nasal provocation test (NPT). Additionally, we evaluated if other factors like being in contact with pets could be associated with pet sensitization and respiratory symptoms.

## 2. Methodology

### 2.1. Study Design

This is a cross-sectional study of cases and controls, with two principal aims. The first one is to compare the frequency of sIgE sensitization to different dander extracts and pet's allergens between a group of patients with rhinitis and a control group; the second one is to evaluate the clinical relevance of pet sensitization according to NPT.

### 2.2. Study Population

Informed consent for children and adults were approved by the institutional and ethics committees of the University of Antioquia and “IPS Universitaria” Clinic (Medellín, Colombia). Patients over six years old were selected from a population of individuals who were diagnosed with rhinitis and attended to the allergy service for skin prick test (SPT). Disease diagnosis for rhinitis and asthma was established according to ARIA guidelines [14, 15] and GINA recommendations (https://www.ginasthma.org). Control subjects were recruited from the University of Antioquia staff and from patient companions that attended the university clinic. The control group consisted of healthy subjects without rhinitis or any other allergy diseases.

### 2.3. IgE Sensitization to Pet Dander and to Other Allergenic Sources

Pet dander sensitization was evaluated according to SPT and serum levels for cat and dog dander extracts. The extracts for SPT were provided by Inmunotek Laboratory (Madrid, Spain). We followed the international recommendations for the SPT [3, 16] and a wheal ≥ 3 mm was considered as positive compared to the negative control.

Serum levels of sIgE for the different molecular components from *Canis familiaris* (Can f 1, Can f 2, Can f 3, and Can f 5) and *Felis domesticus* (Fel d 1, Fel d 2, and Fel d 4) were measured using fluorescence-enzyme immunoassay (Phadia ImmunoCap System, Uppsala, Sweden). The results of serum sIgE were analyzed as a continuum variable (quantitative analysis) and as a categorical variable (qualitative analysis); values equal or above 0.35 KUA/L were considered positive (+) and the ones below that (<0.35 KUA/L) were considered negative (−) as recommended by the manufacturer.

To evaluate atopy with other allergenic sources, according to the prevalence of them in the region, SPT to mites, fungus, insects, and grass was done [1, 17].

### 2.4. Nasal Provocation Test (NPT)

All subjects in both groups with a positive atopy test SPT/sIgE (either to pet dander or molecular components) were called to perform a NPT with pet extract, to confirm the clinical relevance of sIgE sensitization. Patients with sIgE sensitization to more than one animal have two or more provocation tests. NPT was done according to international recommendations [18, 19]. We used subjective (symptoms scale) and objective (acoustic rhinometry) parameters to evaluated nasal provocations tests before and after allergen exposition. Baseline conditions of the nose before performing nasal provocation test was recorded to evaluate if the patients were in optimal conditions to the test and the changes that occurred after the exposure. Standardized extracts were used for nasal provocation (Inmunotek Laboratory), and any medication that could alter the provocation was suspended before the test for at least one week. Fifty microliters of saline solution was applied in each of the patient's nostril using a spray, and the evaluation of nonspecific reactions was done after 30 minutes. Afterwards, the same procedure was done using the extract to be tested, following the manufacturer recommendations, and the patient was kept in observation for one hour. Finally, if the patient did not present any reaction, a tenfold higher concentration was applied and the patient was left under observation for another hour. A positive result was considered according rhinoscopy and clinical evaluation [18].

Prior to use, the presence in the extracts of each of the allergens studied in the serum was evaluated and its concentration measured. Although the concentration of some allergens was variable among the extracts, the concentration was always higher than 2.2 *μ*g/ml and remained in a range between 2.4 and 3.2 *μ*g/ml, which ensures a good representativeness and potency of these allergens for the nasal challenge.

### 2.5. Statistical Analyses

Analyses were performed with IBM SPSS®, version 21, for Windows. For the descriptive analysis of the sociodemographic and clinical aspects of the patients, absolute, relative distributions, and summary indicators were used, such as median, quartiles, interquartile range (IQR), maximum values, and minimum values. The criterion of normality of some clinical variables was established by means of the Shapiro-Wilk test. We used the interquartile range because the variables did not show normal behavior. To establish the relationship between the study groups (rhinitis vs. control) with the characteristics of sensitization to pets, nonparametric tests were applied such as Pearson's chi-squared test of independence, exact Fisher's, or Mann–Whitney *U* test; similarly, the strength of association was evaluated by means of the proportion ratio (PR) with their respective 95% confidence intervals. A *p* value of <0.05 was considered statistically significant.

The same protocol used for dogs and cats was applied in both groups with horse dander, horse serum, and horse allergen molecular components (Equ c 1); however, horse IgE sensitization was low (5 patients, 4.9%) and we were unable to perform additional analyses.

## 3. Results

### 3.1. Population Characteristics

A total of 101 patients with rhinitis and 68 control subjects were included in the study (Table 1). Thirty-five patients with rhinitis also had asthma. Atopy to cat dander but not dog dander was higher in the rhinitis group according to serum sIgE (*p* = 0.05) and SPT (*p* = 0.002).

Dog and cat ownerships were similar between rhinitis and control group (Table 1). In the rhinitis group, 41 (40.5%) patients had a self-report of respiratory exacerbation with dog contact and 20 (29.4%) with cats; when focusing on pet ownership patients, 32% (8 of 25 patients) had a self-report of symptoms with dog (*p* = 0.2) and 40% (6 of 15 patients) with cat (*p* = 0.2), without significant differences with control group. IgE sensitization to other allergenic sources especially mites was common among the rhinitis group (84%) and lower in the control group (18%); 64% of patients with pet sensitization had also mite sensitization.

### 3.2. Sensitization to Dog Dander and Molecular Components

**Figure 1:**
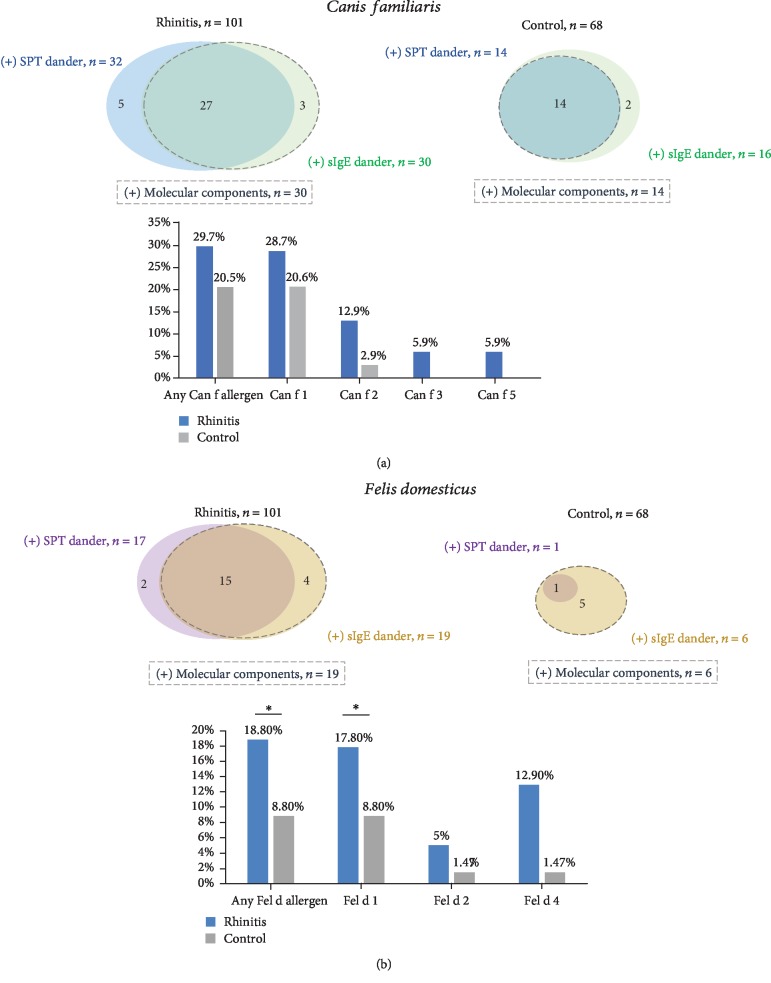
Prevalence of IgE sensitization to cat and dog dander. Concordance of IgE sensitization with dander extract and molecular components are represent in circles. Prevalence of patients with sIgE over 0.35 kUA/L to allergenic components for dog (a) and cat (b) is represented in columns. ^∗^*p* ≤ 0.05.

Between patients and healthy subjects, there was not a significant difference in the frequency of atopy to dog dander according to SPT, sIgE to dog dander (Table 1), or dog components (Figure 1(a)). Patients with sIgE to dog components were all of them positive to sIgE to dog dander, and most of them had a positive SPT with dog dander extract (Figure 1(a)). Sensitization to at least one molecular component of dog was present in 30 (29.7%) patients and 14 (20.5%) healthy subjects (*p* = 0.12) (Figure 1(a)). Sensitization to Can f 1 and Can f 2 was higher in the rhinitis group, but without statistically significant difference when compared with the control group (Figure 1(a)). Only the rhinitis group had sIgE sensitization to Can f 3 and Can f 5.

**Figure 2:**
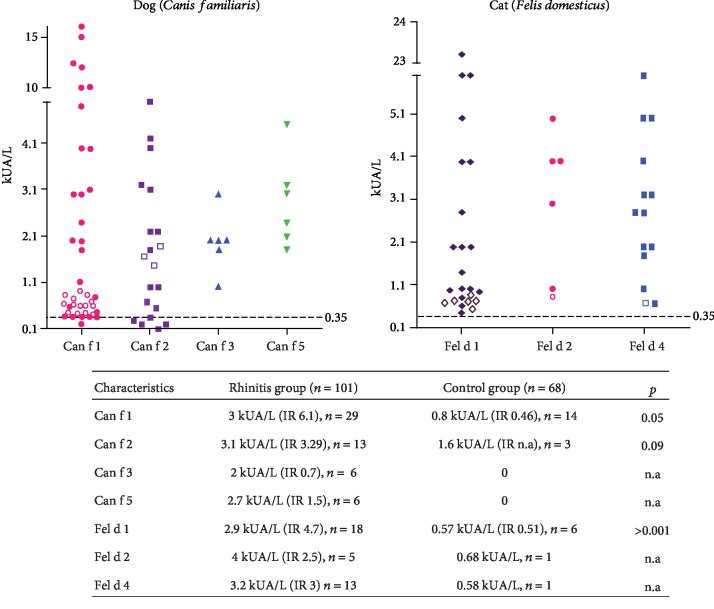
Concentration of dog and cat allergenic components. Patients from the rhinitis group (stuffed) and control group (empty) with sIgE to molecular components. The median and interquartile range (IQR) for each allergenic component among subjects with (+) sIgE over 0.35 kUA/L. n.a: not applicable.

**Figure 3:**
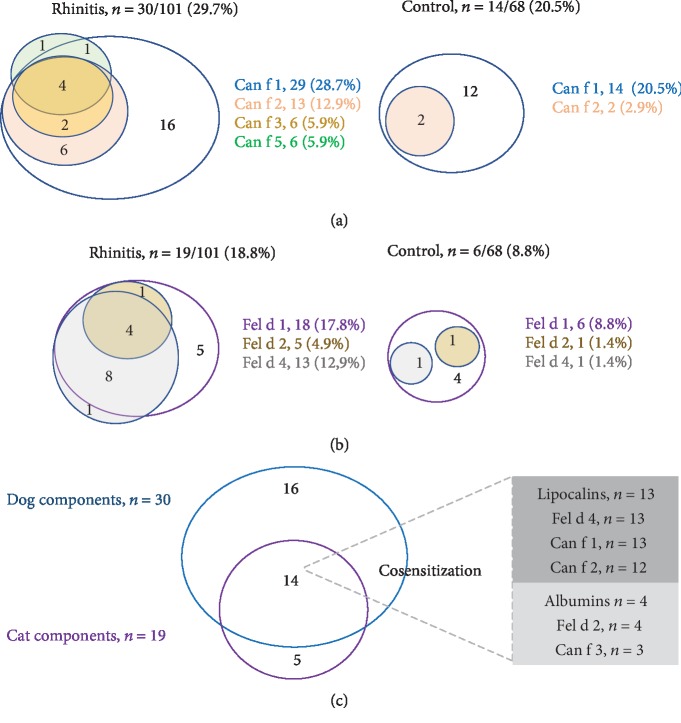
Interaction of cat and dog components. The number of patients or control subjects with (+) IgE (≥0.35 kUA/L) to each component from dogs (a) and cats (b) is represented in circles with different sizes. Cosensitization of cat and dog components is presented in (c).

The concentration of sIgE to Can f 1 among the (+) sIgE subjects was higher in the rhinitis group (*p* = 0.05) (Figure 2). Polysensitization to two or more dog components was more frequent in the rhinitis group than in the control group (15 of 30 patients (50%) vs. 3 of 14 healthy subjects (21.4%), *p* = 0.05) (Figure 3(a)).

Dog ownership was similar in the rhinitis and control groups (Table 1), but rhinitis subjects with dog ownership had a higher concentration levels of sIgE to Can f 1 (*p* = 0.03) and Can f 2 (*p* = 0.04) (data not shown). The frequency of dog ownership among patients with (+) sIgE to Can f 3 was 66%, the same as that for patients with (+) sIgE to Can f 5.

In the rhinitis group, patients with asthma had a higher sensitization to Can f 1 (54.2% vs. 15.1%, *p* < 0.001), Can f 2 (22.8% vs. 7.5%, *p* = 0.05), Can f 3 (11.4% vs. 3%, *p* = 0.01), and Can f 5 (11.4% vs. 3%, *p* = 0.01) than patients without asthma. There was a strong relationship among SPT and sIgE results.

### 3.3. Sensitization to Cat Dander and Molecular Components

The number of patients with cat dander atopy was higher in the rhinitis group than in the control group according to SPT or sIgE (Table 1). Sensitization to at least one cat component was present in 19 (18.8%) patients from the rhinitis group and 6 (8.8%) in the control group (*p* = 0.05). Positive sIgE to Fel d 1 but not to Fel d 2 and Fel d 4 was significantly higher in the rhinitis group (Figure 1(b)). From both groups, rhinitis patients with (+) sIgE to Fel d 1 had higher concentration levels than control subjects (Figure 2). Polysensitization to cat components was higher in the rhinitis group (Figure 3(b)).

Cat ownership was similar in rhinitis and control groups (*n* = 15, 14.9% vs. *n* = 12, 17.6%, respectively). Among ownership subjects, levels of Fel d 1 were higher in patients with cat ownership than that in the control group (data not shown). In the rhinitis group, patients with asthma had a higher sensitization to cat components than in patients without asthma; Fel d 1 (31.4% vs. 10.6%, *p* = 0.009), Fel d 2 (11.4% vs. 1.5%, *p* = 0.04), and Fel d 4 (25.7% vs. 6%, *p* = 0.01).

Fourteen of the nineteen (73.6%) patients with sensitization to at least one cat allergen had cosensitization with dog allergens (Figure 3(c)). From the lipocalin components, thirteen of the fourteen (92.8%) patients with cosensitization were sensitize to Fel d 4; all of them had (+) sIgE to Can f 2 and 12 (85.7%) to Can f 1. From the albumin components, four of the fourteen (28.5%) patients with cosensitization had (+) sIgE to Fel d 2 and three of them (75%) to Can f 3.

### 3.4. Evaluation of the Clinical Relevance of IgE Sensitization to Dog

**Figure 4:**
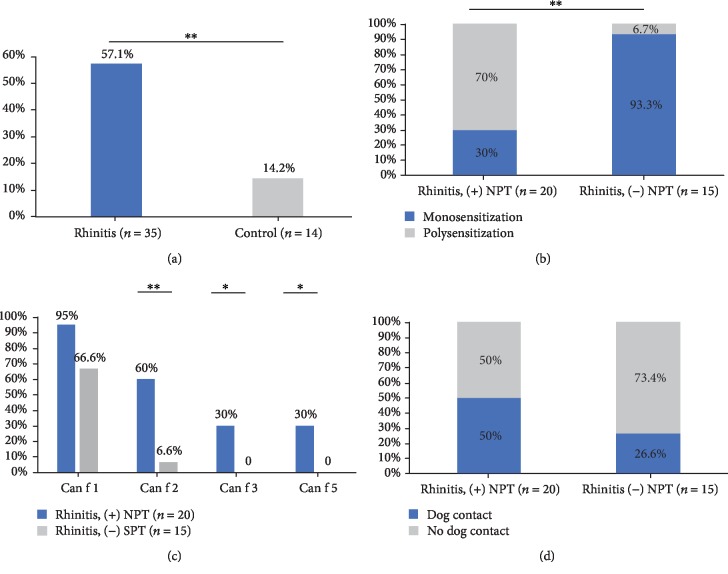
Nasal provocation test with dog components. Positive nasal provocation test (NPT) results were higher in the rhinitis group (a). Patients sensitized with Can f 2, Can f 3, or Can f 5 and (+) NPT had cosensitization with Can f 1 (b). Poly and monosensitization according NPT results are represented in (c). Panel (d) represents the NPT results according dog contact. ^∗^*p* ≤ 0.05, ^∗∗^*p* ≤ 0.01.

Subjects with atopy to dog dander or any dog component underwent NPT with dog extract (thirty-five patients and sixteen control subjects; two control subjects did not accept NPT). Positive NPT was higher in the rhinitis group than in the control group (*p* = 0.01). In the control group, two subjects had a (+) NPT with dog extract (Figure 4(a)); both subjects were sensitized to Can f 1 and one of them also to Can f 2.

Sensitization to pet dander (*p* = 0.12), or with Can f 1 (*p* = 0.06), had no significant association with (+) NPT but there was a higher concentration of sIgE to Can f 1 among patients with (+) NPT (median: 4.9, IQR: 7 vs. 0.39, IQR: 0.05, *p* < 0.001). Among the rhinitis group, polysensitization with Can f 1 and any additional dog components had a significant association with (+) NPT (Figures 4(b) and 4(c)). The ration of proportion for polysensitization as a risk factor to (+) NPT was 3.1 (95% CI: 1.5 to 6.1, *p* < 0.001).

Dog ownership (Figure 4(d)) or asthma was not associated with (+) NPT in the rhinitis or control group.

### 3.5. Evaluation of the Clinical Relevance of IgE Sensitization to Cat

**Figure 5:**
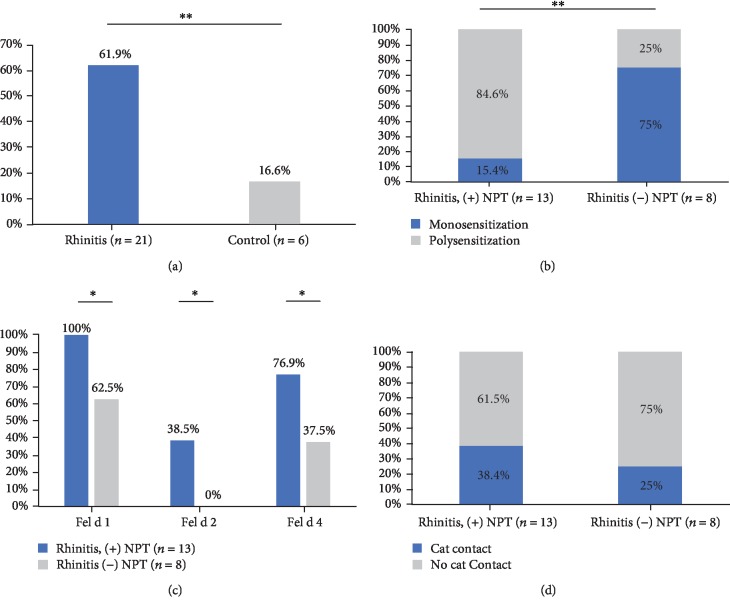
Nasal provocation test with cat components. Positive nasal provocation test (NPT) results were higher in the rhinitis group (a). Patients sensitized with Fel d 2 and Fel d 4 and (+) NPT (b) had cosensitization with Fel d 1. Poly and monosensitization according NPT results are represented in (c). Panel (d) represents the NPT results according cat contact. ^∗^*p* ≤ 0.05, ^∗∗^*p* ≤ 0.01.

Patients (*n* = 21) or control subjects (*n* = 6) with a positive test to cat dander (SPT or serum test) or any cat component underwent NPT with cat extract. Positive NPT was higher in the rhinitis group than in the control group (*p* = 0.01) (Figure 5(a)).

Sensitization to pet dander (*p* = 0.07) had no significant association with (+) NPT. Patients with sIgE to Fel d 1, especially those with high concentrations (three quartile), had a higher probability to have a (+) NPT (Figure 5(b)) (median: 4.0, IQR: 5 vs. 1.4, IQR: 1.8, *p* = 0.026). This probability was higher when the patient had sIgE sensitization to Fel d 1 and any other additional cat components (Fel d 1 and others) (Figures 5(b) and 5(c)). The ration of proportion for polysensitization as a risk factor to (+) NPT was 2.5 (95% CI: 0.8 to 8.0, *p* = 0.01).

Cat ownership was not associated with NPT results in any group (Figure 5(d)). Eight of thirteen patients with (+) NPT had asthma, but it was not a statistically significant factor for a (+) NPT (*p* = 0.3).

## 4. Discussion

Allergies to furry animals affect the population worldwide and is a growing public health concern [20]. The highest densities of pets are found in metropolitan areas [21], and allergens from furry animals are encountered widely in public places [22]. Indeed, the presence of pet allergens has been demonstrated in schools, in day-care centers, on public transport, and in households of non-pet owners [23, 24]. Prevalence of sIgE sensitization to dogs and cats changes according to the evaluated population [20] but also according to the diagnostic technique used to evaluate atopy [25]. We observed that evaluation of cat and dog atopy, using pet dander extracts in SPT and serum test, was similar than molecular components in the rhinitis and control groups, but dander extracts were not specific enough to predict positive NPT to dog or cat adequately. Therefore, additional tools are required to identify patients with clinically relevant atopy to furry animals.

Stokholm et al. [8] noted that extreme exposure to cats (but not dogs) could protect against the development of respiratory symptoms. However, Collin et al. [26] found that pets including cats and dogs could be a risk factor for a nonatopic asthma. According to our results, having a cat or dog was not a significant risk factor of atopy or positive NPT; perhaps because even in subjects without pets in homes, indirect contact with pet allergens is high. These apparently contradictory results indicate that each population have specific factors [22], which may influence the onset of atopy, and if it is clinically relevant. Horseback riding in some populations is a common leisure activity [27], but contact with horse in our population was low, which could explain the low frequency of sensitization (data not shown).

Similar to previous studies, we observed that Can f 1 and Fel d 1 are the most prevalent allergens from dogs and cats in the rhinitis group and in healthy population. The high sensitization to Can f 1 and Fel d 1 can be explained by the fact that these are proteins abundantly produced by pets and their contact with humans is facilitated by their ability to move in the air [6, 28].

We observed a high cosensitization between Can f 1 and Can f 2 in the rhinitis group. Likewise, the cross-reactivity with lipocalins and albumins from different species could explain the high frequency of atopy to dogs among patients with cat sensitization, but cross-reactivity does not explain the high cosensitization among Can f 1 (lipocalin) and other dog allergens like Can f 3 (albumin protein) and Can f 5 (prostatic kallikrein). Also, most patients sensitized to Fel d 2 (albumin) and Fel d 4 (lipocalin) were sensitized to Fel d 1 (uteroglobin), being the three components from different protein families. We believe that in allergic patients, due to their proinflammatory state and/or the lack of counter-regulatory mechanisms, the initial sIgE sensitization to Can f 1 and Fel d 1 (the most frequently found and in higher concentration levels) favors IgE sensitization to other molecular components; this hypothesis is supported by the observation that sensitization to Can f 3 and Can f 5 was exclusive in the rhinitis group, and they had a higher frequency of sensitization to Fel d 2 and Fel d 4. In addition, mechanisms not associated with cross-reactivity, like tissue damage and enzymatic activity, may contribute with increasing inflammation [29–34].

The association of a particular molecular component from furry animals with specific clinical conditions seems to change between populations [35, 36]. In a Spain population, Can f 3 and Can f 5 had a low prevalence of 9.3% and 33%, respectively, but it was associated with self-report of moderate/severe rhinitis [11]; in a Sweden population, sensitization to Can f 5 was high (61%), but it was not associated with nasal symptoms [37]. The divergent results about the clinical relevance of sensitization to dog or cat components might be explained, in part, by the lack of objective evaluations like provocation test in most of the studies. Käck et al. [37] observed that Can f 5 could be regarded differently in the contexts of monosensitization (low risk for (+) NPT) and polysensitization (high risk for (+) NPT) and other studies perceived a relationship among Can f 5 and Can f 3's monosensitization and respiratory symptoms [11, 12, 38].

We could not explore the clinical relevance of monosensitization to Can f 2, Can f 3, Can f 5, Fel d 2, and Fel d 4 because in our population, almost all patients sensitized to these molecular components were also sensitized to Can f 1 and Fel d 1, respectively. Nevertheless, we found a significant association between (+) NPT and polysensitization. Polysensitization to Can f 1 and any other dog component (Can f 2, Can f 3, or Can f 5) increases the probability for a (+) NPT. In a similar way, polysensitization of Fel d 1 with Fel d 2 or Fel d 4 have a better prognostic index than Fel d 1 alone. Additionally, polysensitization to several components from different animals was a risk factor to have multiple positive nasal provocations; patients with (+) NPT to cat had sensitization to dogs and most of them also have a (+) NPT with dogs. In this scenario, the cross-reactivity to lipocalins and albumins seems to be important, and the fact that the dog was the animal with the highest prevalence of sensitization suggests that it is the primary sensitizer in most of the cases, maybe due to its greater distribution. In the five patients with horse sensitization, the two patients with positive NPT had a positive provocation test with dog and one with cat, but we do not present these results due to the small number of sensitized subjects.

Our study has some limitations. The sample size was calculated according to the frequency of occurrence of the event (people with specific IgE to an allergen) reported in other studies. Interestingly, our population had a much lower sensitization for some allergens, which limited the extent of our analyses. However, the calculated power for the available sample was greater than 80% despite the lower incidence of the study event. Only 35 patients with varying sensitization patterns to both cats and dogs are the main drawback and limit the possibility to subgroup analysis. Nevertheless, the source population was large (*n* = 101), so the low number of patients sensitized to several sources adequately reflects the sensitization pattern of this population. Extracts for nasal provocation tests and SPT could differ significantly in the content of allergenic proteins, and such a difference for instance could induce information bias. To ensure the relevance of each component, it would be necessary to perform an individual nasal test with each one, which was not possible for us and is usually not practical in the clinic. However, in the extracts used in the provocation test, the minimum concentration of each component studied was 2.2 *μ*g/ml, and it remained in a range between 2.4 and 3.2 *μ*g/ml, which ensures a good representativeness and potency of these allergens for nasal challenge. Additionally, all tested molecular components of both cats and dogs have expression in the epithelium of pets which decreases the probability that it is not found in the extract used.

We observed differences in the pattern of sensitization between patients with rhinitis only and rhinitis with asthma, but the presence or absence of asthma does not seem to be an additional risk factor for a (+) NPT to cats or dogs. Additional studies comparing the pattern of patients with only asthma could clarify if these differences in allergen sensitization have a clinical relevance or not.

In conclusion, we observed that sIgE sensitization to pet dander identifies most atopic patients, but its utility to predict clinical relevance is unclear. Diagnosis of polysensitization to pet components is a useful tool to predict clinical relevance in rhinitis patients. The sIgE cross-reactivity to lipocalins and albumins seems to explain the high cosensitization to dogs among patients sensitized to cat and horse, but it is necessary to perform studies with a larger sample size of patients, additional animals, and include other allergens to confirm these results. The clinical implications of the different patterns of sensitization in immunomodulatory treatment should also be studied.

## Figures and Tables

**Table 1:** Population characteristics.

Characteristics	Rhinitis group (*n* = 101)	Control group (*n* = 68)	*p*
Mean age in years	20 (median: 17, IQR: 5 to 60)	18 (median: 12, IQR: 6 to 54)	>0.05
Sex: female, *n* (%)	46 (45%)	34 (50%)	>0.05
Asthma, *n* (%)	35 (34%)	0	<0.001
Dog ownership	25 (24.8%)	24 (35.3%)	>0.05
Cat ownership	15 (14.9%)	12 (17.6%)	>0.05
Positive SPT to any animal	33 (32.7%)	14 (20.6%)	>0.05
Positive SPT dog dander (%)	32 (31%)	14 (20%)	>0.05
Positive SPT cat dander (%)	17 (16.8%)	1 (1.5%)	0.002
(+) IgE dog dander, *n* (%)	*n* = 30 (29.7%)	*n* = 16 (23.5%)	>0.05
(+) IgE cat dander, *n* (%)	*n* = 19 (18.8%)	*n* = 6 (8.8%)	0.05
(+) IgE levels to dog dander, median	5.5 (IQR: 11.7)	1.1 (IQR: 1)	0.02
(+) IgE levels to cat dander, median	4 (IQR: 10)	0.8 (IQR: 0.6)	0.001

Population characteristics and sensitization to pet dander according skin prick test (SPT) and serum IgE. IQR: interquartile range.

## Data Availability

The data used to support the findings of this study are available from the corresponding author upon request.
